# Computational Interspecies Translation Between Alzheimer’s Disease Mouse Models and Human Subjects Identifies Innate Immune Complement, TYROBP, and TAM Receptor Agonist Signatures, Distinct From Influences of Aging

**DOI:** 10.3389/fnins.2021.727784

**Published:** 2021-09-30

**Authors:** Meelim J. Lee, Chuangqi Wang, Molly J. Carroll, Douglas K. Brubaker, Bradley T. Hyman, Douglas A. Lauffenburger

**Affiliations:** ^1^Department of Biological Engineering, Massachusetts Institute of Technology, Cambridge, MA, United States; ^2^Weldon School of Biomedical Engineering, Purdue University, West Lafayette, IN, United States; ^3^Regenstrief Center for Healthcare Engineering, Purdue University, West Lafayette, IN, United States; ^4^MassGeneral Institute for Neurodegenerative Disease, Massachusetts General Hospital, Boston, MA, United States

**Keywords:** Alzheimer’s disease, transcriptomics, systems biology, cross-species analysis, TAM receptor agonists

## Abstract

Mouse models are vital for preclinical research on Alzheimer’s disease (AD) pathobiology. Many traditional models are driven by autosomal dominant mutations identified from early onset AD genetics whereas late onset and sporadic forms of the disease are predominant among human patients. Alongside ongoing experimental efforts to improve fidelity of mouse model representation of late onset AD, a computational framework termed Translatable Components Regression (TransComp-R) offers a complementary approach to leverage human and mouse datasets concurrently to enhance translation capabilities. We employ TransComp-R to integratively analyze transcriptomic data from human postmortem and traditional amyloid mouse model hippocampi to identify pathway-level signatures present in human patient samples yet predictive of mouse model disease status. This method allows concomitant evaluation of datasets across different species beyond observational seeking of direct commonalities between the species. Additional linear modeling focuses on decoupling disease signatures from effects of aging. Our results elucidated mouse-to-human translatable signatures associated with disease: excitatory synapses, inflammatory cytokine signaling, and complement cascade- and TYROBP-based innate immune activity; these signatures all find validation in previous literature. Additionally, we identified agonists of the Tyro3 / Axl / MerTK (TAM) receptor family as significant contributors to the cross-species innate immune signature; the mechanistic roles of the TAM receptor family in AD merit further dedicated study. We have demonstrated that TransComp-R can enhance translational understanding of relationships between AD mouse model data and human data, thus aiding generation of biological hypotheses concerning AD progression and holding promise for improved preclinical evaluation of therapies.

## Introduction

Alzheimer’s disease (AD) is the most common form of dementia, characterized by loss of cognitive functions such as memory and the presence of hallmark amyloid and tau histopathologies in the brain. AD is associated with aging, with the majority of patients presenting with disease after the age of 65. In 2018, there were approximately 30 million diagnosed cases of AD worldwide, with 82 million cases projected by 2030 (von Schaper, 2018). The projected increase in cases is, in part, indicative of the increase of AD prevalence coming about with increased age of the global population. Understanding of disease progression is currently limited, and the majority of existing, FDA-approved therapies for AD only provide symptomatic relief (von Schaper, 2018).

AD mouse models are an important piece of both research toward understanding AD pathobiology and preclinical evaluation of resulting therapies. However, there are limitations with the existing AD mouse model landscape. The majority of existing AD mouse models are based on mutations identified in Early Onset Alzheimer’s Disease (EOAD). EOAD refers to subtypes of AD that present before the age of 65. Most EOAD cases can also be classified as familial or dominant inheritance forms of the disease that commonly involve an aggressive mutation(s) along the hallmark amyloid cascade to account for disease onset. Examples of relevant amyloid mouse model strains include the TAS10 (*APP* KM670/671NL), TPM (*PSEN1* M146V), TASTPM (*APP*_*swe*_, *PSEN1* M146V) and 5XFAD (*APP*_*swe*_, *APP* I716V, *APP* V717I, *PSEN1* M146L, *PSEN1* L286V) models (Richardson et al., 2003; Howlett et al., 2004; Oakley et al., 2006). Additionally, there are mouse models such as the 3xTg (*APP*_*swe*_, *MAPT* P301L, *PSEN1* M146V) model which combine amyloid and tau mutations (Oddo et al., 2003). Each of these individual mouse models incompletely recapitulates known AD outcomes. For example, amyloid precursor protein (APP) models demonstrate amyloid plaque deposition and peri-plaque synapse disruption, but there is generally a lack of other critical features of the disease such as neurofibrillary tangle (NFT) formation and loss of synapses and neurons. Tau models show aggregated mutant tau, which has a different conformation than the wild type tau that makes up Alzheimer tangles, and neuronal loss but an absence of amyloid plaque deposition.

Amyloid and tau are necessary features of AD, and there is utility resulting from the decoupled amyloid and tau phenomena in amyloid-specific and tau-specific mouse models. However, the clinical reality is that EOAD comprises only a very small percent of human AD cases (Holtzman et al., 2011). The more common forms of AD are classified as late onset (after the age of 65) and sporadic, meaning that there are not immediately clear autosomal dominant mutations driving disease. A major patient risk factor of late onset AD (LOAD) is considered to be aging itself (De Strooper and Karran, 2016; Hargis and Blalock, 2017).

To further complicate the utility of amyloid and tau mouse models, previous literature has shown that the presence of amyloid plaques and NFTs are insufficient to completely account for clinical symptoms of cognitive decline. Plaques correlate quite poorly with cognitive scores; tangles and their distribution are more closely correlated with both neuronal loss and cognitive impairment, but there remains a good deal of unaccounted cognitive decline even after taking tangles into account. In some instances, Perez-Nievas et al. (2013) identified resilient individuals who were histopathologically positive for substantial amyloid plaques (10D5 antibody) and tau tangles (PHF-1 antibody) but negative for clinical symptoms of dementia. The authors found that other markers such as glial activation as measured by GFAP- and CD86-positive staining better correlated with clinical outcomes.

There are continued experimental efforts to improve the fidelity of AD mouse models. For example, Cruz et al. (2003) developed the CK-p25 mouse, a model with inducible over-expression of p25 that drives Cdk5 kinase dysregulation and changes in APP processing. There are also multiple AD mouse models based on high risk genetic factors identified through genome-wide association studies (GWAS) for late onset forms of AD. For example, Model Organism Development and Evaluation for Late-onset Alzheimer’s Disease (MODEL-AD) is an effort to generate more publicly available AD mouse models. They have utilized recent genetic studies suggesting that minor alleles in dozens of genes contribute to risk of AD. To date, the consortium has developed mouse models carrying variants of *ABCA7*, *APOE* (e.g., e4), *CEACAM1*, *IL1RAP*, *PLCG2*, and *TREM2* to better mimic human LOAD (Reardon, 2018; Oblak et al., 2020).

Synergizing with novel mouse model development, there are experimental efforts to better characterize the effects of diverse genetic backgrounds on EOAD mutation effects. In Neuner et al. (2019), the authors generated F1 hybrids by crossing the EOAD-mutation based 5XFAD mouse with the BXD reference panel of mice. The resulting F1 mice were referred to as AD-BXD mice (Neuner et al., 2019). The authors focused on characterizing the phenotypic variability that resulted from subtle difference in genetic background. For example, the authors identified resilience toward AD transgenes in C57BL/6J background mice. The authors work points toward understanding the effects of human genetic variability on AD risk, development, progression, and presentation.

Computation offers additional avenues to address strengths and limitations of existing AD mouse models via cross-species analysis. There is considerable previous work applying computational analysis toward AD datasets (Miller et al., 2010; Zhang et al., 2013; Burns et al., 2015; Friedman et al., 2018; Mostafavi et al., 2018; Wan et al., 2020). Here, we will discuss studies that specifically combine mouse model and human postmortem datasets and highlight studies employing different strategies for cross-species analysis. In Burns et al. (2015), the authors identified mouse model datasets across multiple neurodegenerative diseases and compared gene set enrichment analysis (GSEA) results from each dataset to that of human datasets for AD, Parkinson’s Disease, Huntington’s Disease, and Amyotrophic Lateral Sclerosis. The observational comparison found that gene set enrichment signatures were shared across human neurodegenerative disease datasets but poorly translated between mouse and human analyses. In Friedman et al. (2018), the authors leveraged the increased granularity accessible with mouse model transcriptomics in their cross-species analysis. The authors used microglia and myeloid-specific mouse datasets spanning AD and other acute and chronic CNS disease states. They first applied statistical meta-analysis, specifically hierarchical clustering of differentially expressed genes, to identify mouse-specific modules and then evaluated bulk human transcriptomic data for these signatures at the level of orthologous genes with mixed results. In a recently published resource, Wan et al. (2020) conducted a meta-analysis aggregating publicly available human and mouse datasets spanning various neurodegenerative diseases and brain regions in a species specific fashion. The authors applied multiple co-expression methods including WGCNA to identify human-specific modules. Mouse datasets were analyzed for differential gene expression comparisons. The authors identified human modules that overlapped with mouse analysis across AD and other neurodegenerative diseases, and modules were enriched for neuronal genes and microglial genes.

In this study, we aimed to conduct cross-species analysis that leverages existing mouse model datasets by modeling human and mouse data concomitantly in integrated manner. We employed an interspecies analysis method previously developed in our group, Translatable Components Regression (TransComp-R; Brubaker et al., 2020). Using TransComp-R, we concurrently analyzed transcriptomic data from human postmortem brain and traditional amyloid AD mouse model hippocampi to identify pathway-level signatures present in human patient samples yet predictive of mouse model disease status. Additionally, we incorporated linear modeling to discern the effects of disease status from aging. Our workflow is designed to quantitatively identify synergistic enrichment across mouse and human datasets that might not be identified when observationally seeking direct commonalities between the species.

TransComp-R analysis of amyloid mouse model and postmortem human hippocampal data resulted in effective model building and identification of mouse-to-human translatable signatures associated with disease status. Specifically, we identified signatures associated with excitatory synapses, inflammatory cytokine signaling, and complement cascade- and TYROBP-based innate immune activity. We were able to validate these four signatures in previous AD literature. We also noted that mouse model genetics can confound statistical enrichment of signatures that requires careful delineation between mathematical and biological enrichment, as seen during follow-up analysis of the inflammatory cytokine signaling signature. We also identified agonists of the Tyro3 / Axl / MerTK (TAM) receptor family as important contributors to the complement cascade and innate immune signatures; the TAM family of receptors and ligands is lesser studied in the AD literature. Ultimately, we demonstrated the translational utility of TransComp-R for identification of cross-species signatures, its value toward generating biological hypotheses in the context of AD progression, and its potential for rational selection of multiple AD mouse models during preclinical evaluation of therapies.

## Materials and Methods

### Dataset Selection and Processing

#### Publicly Available Dataset Selection

Human postmortem brain tissue and mouse model transcriptomic data used in this study were from publicly available datasets. We obtained the datasets from the Gene Expression Omnibus (GEO), and the specific datasets accession numbers were GSE1297 (human), GSE48350 (human), and GSE64398 (mouse) (Edgar et al., 2002; Blalock et al., 2004; Berchtold et al., 2008, 2013; Cribbs et al., 2012; Matarin et al., 2015).

When selecting datasets, we first searched GEO for bulk transcriptomic datasets from human postmortem brain tissue and AD mouse models. We used matching brain region, mouse sample number, sequencing methodology, and sequencing platform as criteria for evaluating datasets available through GEO. The ultimate selection of the two human and one mouse dataset was based on the presence of samples from the hippocampus across all three studies, the use of microarray as the common sequencing technique, the availability of multiple mouse model samples on the same microarray sequencing platform, and higher sample number per group relative to other studies satisfying similar other criteria.

For GSE1297, there were 31 total samples submitted by the original study. Categorical disease status (control, incipient, moderate, and severe), neurofibrillary tangle score, Braak score, age, Mini-Mental State Examination score, sex and, post mortem interval information was provided for each patient. For GSE48350, there were 253 samples submitted by the original study. The samples spanned the postcentral gyrus, the superior frontal gyrus, the hippocampus, and the entorhinal cortex. Our study used 62 samples specific to the hippocampus. Binary disease status, sex, and age information were provided for each patient. For GSE64398, there were 333 samples submitted by the original study. The samples spanned the cerebellum, cortex, and hippocampus and included wild-type, TAS, TPM, heterozygous TASTPM, homozygous TASTPM, and Tau P301L mice. For this study, we selected hippocampal samples from wild-type, heterozygous TASTPM, and homozygous TASTPM mice. We selected the TASTPM mouse based on greater sample number and more pronounced histopathological progression with age as compared to the other three mouse models included in the original study. Genotype, sex, and age (2, 4, 8, 18-months) information were provided for each mouse.

#### Data Pre-processing and Normalization

We downloaded the raw gene expression data for each study and normalized the datasets via robust multichip averaging. In the case of GSE48350 and GSE64398, we conducted normalization using only hippocampal samples and not all samples available in the original studies; the full datasets included samples from other brain regions (e.g., entorhinal cortex, postcentral gyrus, superior frontal gyrus). We retrieved data using GEOquery and conducted initial data processing using affy and beadarray (Gautier et al., 2004; Davis and Meltzer, 2007; Dunning et al., 2007).

#### Differential Gene Expression Analysis

Following normalization, we evaluated human study GSE48350 for differential gene expression to generate a permissive list of differentially expressed genes (DEGs) for subsequent principal component analysis (PCA). Genes were considered to be in the list of DEGs if they had a Benjamini-Hochberg corrected *p*-value less than 0.20. We did not use fold change criteria because genes with a subtle difference between disease and control groups in one species might have a more pronounced trend in the other, a potentially biologically meaningful relationship we wanted to capture with our model. GSE48350 phenotypic and clinical outcome data provided with the study included age, sex, and a binary disease identifier (i.e., control or AD). As a result, we included age, sex, and binary disease status in the model for DEG evaluation and selected genes based on their differential expression in disease.

#### Human and Mouse Homolog Evaluation

The cross-species analysis workflow employed combines mouse gene expression with human gene loadings from PCA. This requires that all human genes included in the PCA model have mouse homologs. As a result, we filtered for human-to-mouse homologs prior to building the human PCA models using the Mouse Genome Informatics database and the same homolog conversion approach described in Kumar et al. (2018) (Blake et al., 2017). In the case that multiple probes mapped to a gene within a given species, the median expression across all probes for the gene was used.

### Translatable Component Regression

#### Brief Overview of the Translatable Components Regression Workflow

TransComp-R is a method for concomitant evaluation of two different datasets that was previously developed in the lab (Brubaker et al., 2020). Code from the initial study is available via MATLAB File Exchange^1^, and the TransComp-R-specific analyses in this study were also conducted using MATLAB (2018b). We will describe the method here with a focus on describing (1) changes to the workflow that were unique to this study and (2) where the human and mouse datasets were utilized within TransComp-R, since the workflow is directional.

To briefly describe the workflow, we first used a human dataset to generate a PCA model. Then, we projected a mouse dataset into the human PCA space. This combined the two cross-species datasets. The mouse sample scores along the human principal components (PCs) were then regressed against the disease status of the mouse samples. The regression step involved LASSO feature selection followed by linear regression to identify human model PCs predictive of mouse disease outcomes. These PCs were considered to be cross-species translatable components and were the focus of follow-up pathway analysis and biological interpretation.

#### Principal Component Analysis

PCA was conducted to generate a separate model for each human dataset. A permissive list of DEGs was used as input for GSE48350, and all genes with human-to-mouse homologs were used as input for GSE1297. Prior to model construction, each study was internally normalized by z-score. To avoid overfitting, the final PCA models were capped to fewer PCs than the original models. We selected the number of PCs in the final models based on the more restrictive of two criteria – (1) the number of PCs that cumulatively explained at least 95 percent of the total percent variance of the model or (2) only PCs that individually each explained at least one percent of the total percent variance of the human model.

#### Linear Model Building With Human Dataset Phenotypes

Prior to projecting mouse samples into the human PCA space, we generated linear models to connect the human PCs to the human phenotypes in the original studies. Age, sex, and disease status were analyzed alongside human patient scores from PCA via linear modeling followed by ANOVA analysis (R methods package). A linear model was constructed for each of the human principal components to identify PCs that explained the most variance with regard to each of the three types of patient identifiers included. Disease outcome was binary for GSE48350 and categorical for GSE1297.

#### Projecting Mouse Samples Into Human Principal Component Analysis Space

Mouse samples were projected into the existing human PCA space, leveraging homolog matches. Prior to projection, the gene expression for the mouse dataset was internally normalized via z-scoring. Separate normalization is necessary as the human and mouse datasets possess differences including but not limited to species and sequencing platform. This normalization approach allows for us to evaluate relative separation of mouse samples in a PCA space generated using data from a different species.

#### Translatable Component Selection

We sought to identify translatable components by evaluating the relationship between mouse samples scores along human PCs and the mouse sample outcomes. This was a two-step process. First, we conducted LASSO feature selection using a workflow similar to Ackerman et al. (2018) to regress mouse sample scores on human PCs against mouse outcomes to reduce the number of human PC features (Ackerman et al., 2018). We partitioned the data for 5-fold cross validation using cvpartition (MATLAB), which divides the data into k-folds with representation of each phenotype class in each fold when possible. Within each fold, we conducted LASSO ten times using features (i.e., mouse scores on human PCs) from only the training set samples. At the end of 50 runs of LASSO using different subsets of the data, human PCs that were selected in at least 40 percent of runs were utilized for linear model building. In linear model building, only PCs that were selected via LASSO were included for the final model. Because of this model criteria, LASSO determined the number of human PCs that were ultimately included in the final translatable model, and the number of PCs in the final model could and did differ between different mouse-to-human case studies. In order to generate an evaluative metric for the final model, we conducted leave-one-out cross validation and calculated root mean square error (RMSE) using the predicted mouse outcome for each sample when it was held out as the test data.

Translatable component selection requires the use of a phenotype vector. Mouse sample outcomes were encoded numerically as follows. The TASTPM mouse samples included both heterozygous and homozygous samples in the original study. As such, wild-type control mice were assigned a zero value, heterozygous TASTPM mice assigned as one, and homozygous TASTPM mice assigned as two.

#### Evaluating Statistical Significance

The final model was compared against two types of null models to determine the statistical significance of the results. First, we generated random PC models. In this type of null model, we randomly selected a size-matched set of PCs and evaluated the RMSE of linear models built using the randomly selected PCs. We fixed the number of PCs to be identical to the true model, and, at most, 1000 null models were generated to evaluate significance. Second, we generated models to test phenotype permutation. In this type of null model, we scrambled the phenotype vector and then went through the full LASSO feature selection and linear model building process described above using the random phenotype vector. Since the phenotype vector is comprised of zeroes, ones, and twos, we evaluated the scrambled phenotype vectors to ensure that each null model started with a unique phenotype vector. We generated 100 null models of this type. Null models were compared to the true model using leave-one-out RMSE values. The value of the true RMSE relative to the distribution of null RMSE values was used to assign the true model an empirical *p*-value.

#### Linear Modeling to Account for Age

The TransComp-R workflow as described thus far is supervised only in the context of mouse disease status. However, the original mouse model study included samples spanning 2, 4, 8, and 18 months of age. We employed linear modeling to select translatable PCs that had disease-relevant signatures distinct from aging. We built models on a PC-by-PC basis to determine whether mouse disease status could explain information encoded in the mouse sample scores along a given PC that was distinct from information already explained by mouse sample age. Practically, this involved building a pair of linear models for each PC. The first model, which we will call the null model, was built using age as the only factor. In the second model, which we call the alternative model, age and disease status were encoded as factors. We then compared the null and alternative models using an F-statistic test.

Mathematically, this workflow can be represented as follows:


$$Null:PC_{ij}∼1++Age_{i}$$

$$Alternative:PC_{ij}∼1+Age_{i}+Disease_{i}$$

$$F=\frac{\left(RSS_{null}-RSS_{alt}\right)/\left(p_{alt}-p_{null}\right)}{\left(RSS_{alt}\right)/\left(N-p_{alt}-1\right)}$$

where *PC*_*ij*_ indicates mouse sample *i*’s value on human PC *j*, *F* indicates the F-statistic from comparing the null and alternative models, *alt* is an abbreviation for alternative model, *RSS* is residual sum of squares, *N* is the number of samples, and *p* is the degrees of freedom.

#### Pathway Enrichment Analyses

Translatable components that were identified via TransComp-R modeling and linear modeling were then evaluated for biological interpretation. For pathway enrichment analysis, we conducted statistical over-representation analysis. We evaluated the enrichment of pathway members in the genes at the top and bottom 20 percent of a translatable PC’s loadings relative to pathway membership in the gene list for the PC as a whole. We started by evaluating each translatable PC for enrichment of a list of 68 broad pathways. The pathways, listed in Supplementary Table 1, included the pre-curated list of 49 Hallmark pathways from MSigDB as well as pathways that implicated a specific central nervous system cell type, amyloid cascade processes, or immune system processes. *P*-values from the Fisher’s exact test for statistical over-representation were Benjamini-Hochberg corrected.

When enriched pathways were identified, they were further evaluated in two ways. First, we generated a follow-up list of more targeted pathways based on the enriched broad pathway to derive more detail regarding the enrichment. Additionally, we looked at the specific genes driving enrichment. Statistical over-representation analyses provided biological interpretation specific to the human model, as the PC loadings themselves do not contain any information derived from the mouse models. As a result, we looked at mouse gene expression of the genes driving statistical over-representation.

For gene-level analysis, we focused on specific genes guided by statistical significance in mouse and therapeutic relevance in human. For statistical significance, we conducted two-way *t*-tests between wild-type, heterozygous TASTPM, and homozygous TASTPM mice and identified genes that had at least one intergroup, significant comparison relative to WT samples following Benjamini-Hochberg correction for multiple hypothesis testing (FDR < 0.05). For therapeutic relevance in human, we evaluate genes with 1-to-1 human-to-mouse homologs using the Universal Protein Resource (UniProt) database (Bateman et al., 2021). We input statistically significant genes via ‘Gene Name’ and filtered the resulting, mapped entries to those that were ‘Reviewed’ and specific to human. We exported results with ‘Subcellular Location’ to identify secreted factors as a proxy for therapeutically relevant receptor-ligand interactions.

## Results

### Interspecies Translation Successfully Separates Mouse Samples in Human Principal Component Analysis Space

We obtained publicly available human hippocampal postmortem brain tissue transcriptomics (GSE48350, GSE1297) and mouse model hippocampal transcriptomics (GSE64398) for cross-species analysis. The publicly available mouse model dataset included transcriptomics for both heterozygous and homozygous mice with *APP* K670N/M671L and *PSEN1* M146V mutations at 2, 4, 8, and 18 months of age (Matarin et al., 2015). The mouse model with *APP* and *PSEN1* mutations was the TASTPM model specifically and demonstrated robust histopathological progression with age. Homozygous TASTPM mice were reported to show amyloid plaque development by 4 months and demonstrated faster histopathological progression of amyloid deposition as compared to heterozygous TASTPM mice in the original study. The TASTPM mouse model samples evaluated in this study are driven by mutations identified in dominantly inherited forms of AD, as a goal of this study was to leverage the existing wider availability of EOAD mouse models as compared to LOAD mouse models.

**FIGURE 1:**
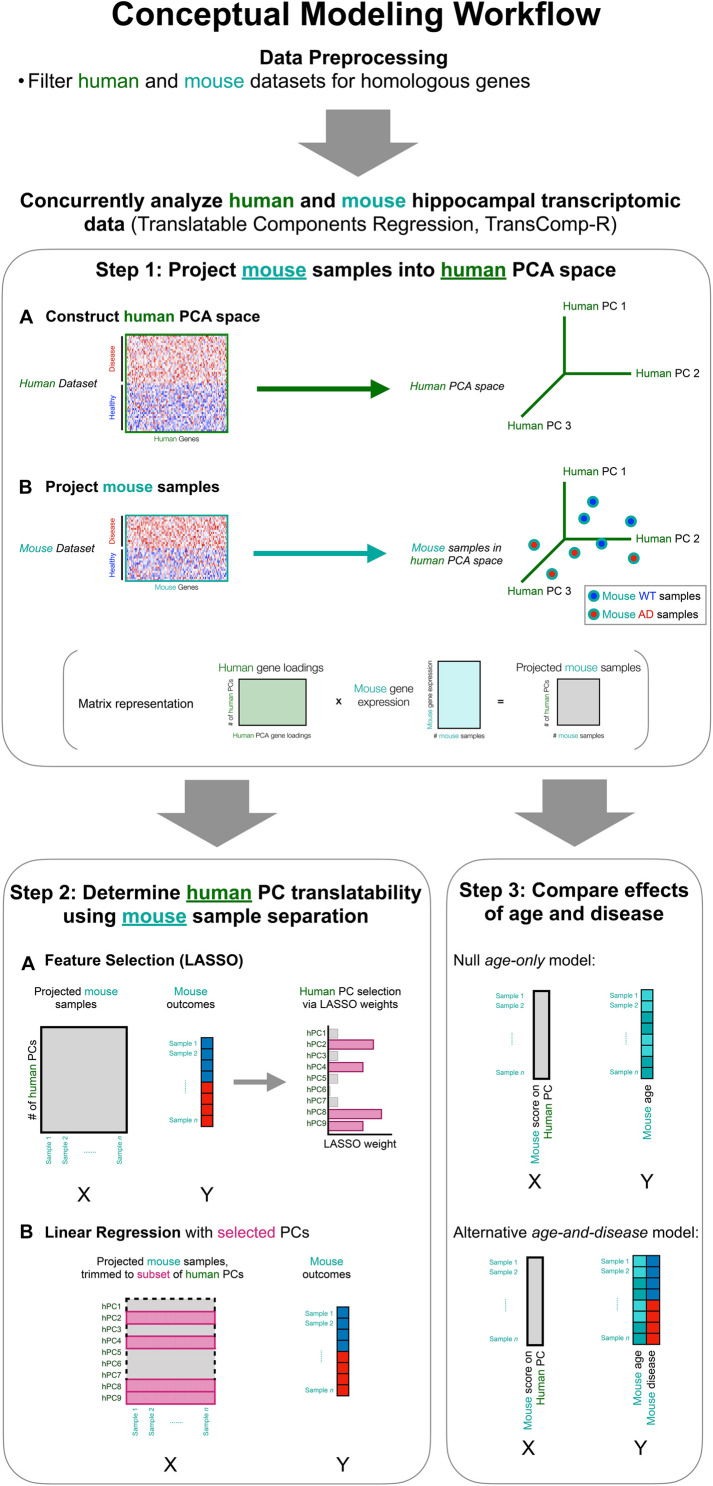
Interspecies translation modeling identifies synergistic molecular signatures that are important in both human and mouse model datasets. The data are pre-processed for normalization and matching of cross-species homologs. The mouse and human data are then combined in a two-step process, which is a modified version of Translatable Components Regression (TransComp-R; Step 1, Step 2). Lastly, the combined data are evaluated to discern the effects of mouse age from mouse disease state (Step 3).

To perform cross-species analysis, we utilized a modified version of the translatable component regression (TransComp-R) modeling methodology (Brubaker et al., 2020). The integrative analysis tool involves a multi-part workflow that is conceptually illustrated in Figure 1. As compared to previous approaches that employed observational comparison between separate human and mouse analyses, TransComp-R allows for concomitant analysis of a human and mouse dataset and leverages synergy across cross-species datasets.

In order to combine a human and a mouse dataset for concurrent analysis, we generated a human principal component analysis (PCA) space and subsequently projected in a mouse dataset (Figure 1, Step 1). We selected this TransComp-R directionality such that we could identify biological signatures present in human patient samples and effective for the separation of mouse samples based on disease status. This translationally points us toward rational selection of multiple disparate mouse models for preclinical evaluation of therapies.

Implementing Step 1 (Figure 1) of the TransComp-R pipeline, we generated human PCA spaces for each of the two publicly available human transcriptomic datasets used in this study. PCA is an unsupervised technique that collapses a high dimensional dataset such that samples can be represented using a smaller set of principal components (PCs). For GSE48350, which will be referred to as human cohort 1 and abbreviated as h1 hence forth, the high dimensional input to PCA was a study-specific list of differentially expressed genes (DEGs). Samples were identified categorically as control or AD in the original study, and DEGs were defined as those different between control and AD patients based on the criteria described in the Methods section. The DEG workflow generated 14,698 features (genes), which is beyond two orders of magnitude greater than the number of hippocampal samples available in the study. As a result, the final DEG list for this study was trimmed to 6,200 genes, on sorting by adjusted *p*-value. Following human-to-mouse homolog matching, there were 4,033 genes.

**FIGURE 2:**
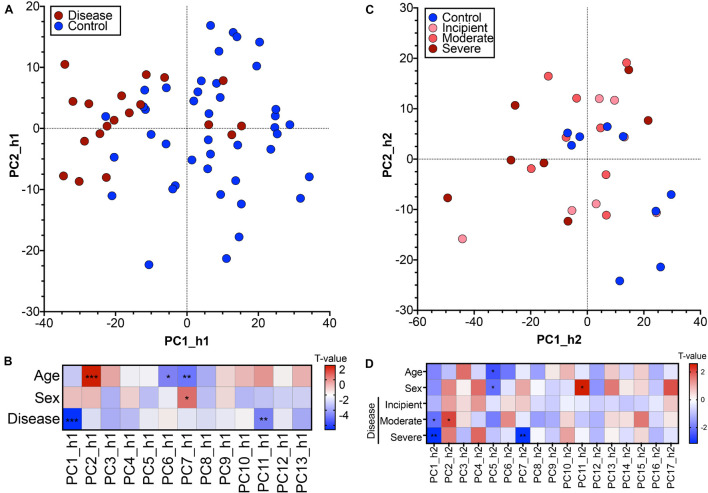
Postmortem hippocampal brain tissue transcriptomics used to generate human Principal Component Analysis models. **(A)** Principal component biplot showing 62 hippocampal human samples (GSE48350, human cohort 1) in PC1_h1, PC2_h1 space. The original, feature-level input to the PCA was genes differentially expressed between control (blue) and disease (red) patients at a permissive threshold of Benjamini-Hochberg adjusted *p*-value < 0.20 with no fold change criteria. **(B)** Linear regression relating human sample scores along each human PC with age, sex, and disease reported for human cohort 1 identified PCs associated with patient outcomes. A positive *t*-value indicates association with higher numerical age and males for the age and sex categories, respectively. **(C)** Principal component biplot showing 31 hippocampal human samples (GSE1297, human cohort 2) in PC1_h2, PC2_h2 space. The original, feature-level input to the PCA was all genes with human-to-mouse homologs. **(D)** Linear regression relating human sample scores along each human PC with age, sex, and disease reported for human cohort 2 identified PCs associated with patient outcomes. A positive *t*-value indicates association with higher numerical age and males for the age and sex categories, respectively. * < 0.05, ** < 0.01, *** < 0.001.

We generated a PCA model for the first human cohort and reduced the dimensionality of the dataset from 4,033 DEGs such that we could represent the 62 human samples along 13 PCs. The 62 human samples are visualized in a biplot along the two highest variance PCs, PC1_h1 and PC2_h1 (Figure 2A). There is loose separation of the samples based on binary disease status along PC1_h1, with disease samples trending toward the left of the PC. The disease relevance of PC1_h1 was confirmed via linear modeling. We constructed a linear model to explain patient sample scores for each of the 13 PCs based on patient age, sex, and disease status. The resulting *t*-values for each of the three patient outcomes are shown alongside the associated ANOVA-derived *p*-values in Figure 2B. In addition to PC1_h1, PC11_h1 is statistically significant with regard to sample disease status information encoded on the PC.

We analyzed transcriptomic data from a second, independent patient cohort (GSE1297), which will henceforth be referred to as human cohort 2 and abbreviated as h2. For the second human cohort, we initialized the PCA space using all genes with human-to-mouse homologs as inputs (8,882 genes). This allowed for a more exploratory analysis compared to the cohort 1 PCA space in which the permissive DEGs introduced a supervised, disease bias in the initial gene input. We reduced the dimensionality of this dataset and generated a PCA model with 17 PCs. The 31 human samples are visualized in a biplot using the two highest variance PCs, PC1_h2 and PC2_h2 (Figure 2C). There is not pronounced separation of the human samples based on disease category on PC1_h2 and PC2_h2, although regression suggests that there is some categorical separation along the PCs (Figure 2D). Linear model building using age, sex, and categorical disease status for each of the PCs additionally identified PC7_h2 as important in disease. There is more pronounced separation of the samples on a categorical basis along PC1_h2 and PC7_h2 (Supplementary Figure 1B).

Having generated human PCA spaces with each of the cohorts, we proceeded to the dataset combination portion of Step 1 of the TransComp-R pipeline and projected the mouse samples into the PCA space (Figure 1, Step 1). Proper normalization of the data is important at this point of the TransComp-R pipeline. Since the mouse model transcriptomics were obtained in a separate study and using a different microarray sequencing platform, we normalized the mouse model data separately prior to projection. As such, Step 1 of the TransComp-R pipeline combines z-scored mouse model expression data with the PCA loading values for each of the corresponding genes from the human data. This keeps the normalization steps separate for each study while still allowing their combination.

Many previous cross-species analyses implement meta-analysis by aggregating datasets on a species-specific basis. We avoid combining datasets within a given species. Instead, we concurrently analyze a single human dataset and a single mouse dataset in their relatively normalized states within each iteration of TransComp-R. This results in separate case studies on a dataset-by-dataset basis and focuses on comparison of biological signature enrichment across cross-species case studies.

**FIGURE 3:**
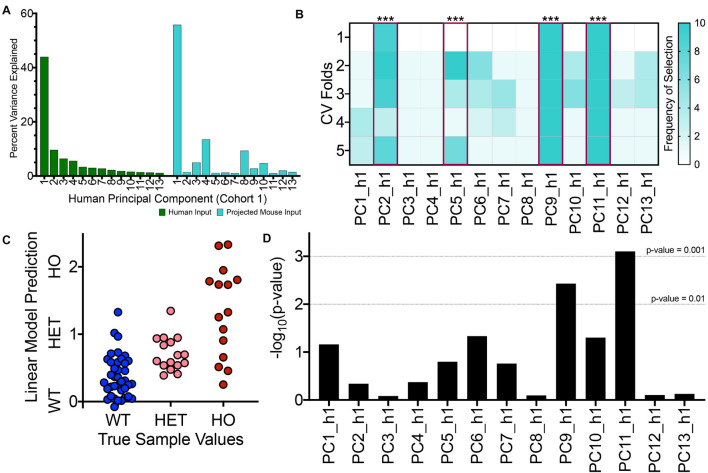
TransComp-R modeling successfully identifies translatable, human principal components that are important for TASTPM mouse outcomes. **(A)** Percent variance explained by each human PC for the original human dataset used to generate the PCA model (GSE48350, cohort 1; green) and the projected mouse dataset (turquoise). **(B)** Frequency of human PC (PC_h1) selection across ten rounds of 5-fold cross validation (CV) using LASSO feature selection based on mouse sample outcome. Human PCs that satisfied the 40 percent selection frequency threshold are highlighted in magenta with the significance of the linear regression model weights provided (*** < 0.001). **(C)** Visualizing linear model predictions on a mouse sample-by-sample basis from the final linear model with four PC inputs across the wild type (WT), heterozygous TASTPM (HET), and homozygous TASTPM (HO) groups. **(D)** Results from linear modeling evaluating which PCs contain information that is relevant for disease-status, in addition to the information encoded by sample age.

As such, we projected samples from the TASTPM mouse and corresponding controls into the PCA space for the first human cohort for our first TransComp-R case study. In Figure 3A, the percent variance explained by each of the 13 human PCs for the original human data is illustrated. The percentage of explained variance decreases with increasing PC number. We also calculated the variance of the matrix of mouse gene expression combined with human PC loadings and determined the percent accounted for by each of the human PCs within the projected matrix. The variance explained by each human PC within the projected mouse matrix does not monotonically decrease with increasing PC number. For example, focusing on PC2_h1, PC3_h1, and PC4_h1, we see that the PCs explain decreasing percent variance for the human dataset and increasing percent variance in the projected mouse matrix. We also see that there are intermediate PCs such as PC8_h1, PC9_h1, and PC10_h1, that explain a sizable amount of projected mouse dataset variance.

Using the projected mouse samples, we proceeded to Step 2 of the TransComp-R workflow to identify translatable human PCs (Figure 1). We undertook a two-step selection process to determine which human PCs encoded information that was both human disease relevant and important for mouse model outcomes. First, we used LASSO feature selection to identify a subset of human PCs that, when combined with mouse sample expression, were important for delineating categorical mouse sample outcome. Specifically, we looked to identify human PCs that effectively separated wild-type (WT) control mice, TASTPM mice that were heterozygous for *APP*_*swe*_ and *PSEN1* mutations (HET), and TASTPM mice that were homozygous for the same mutations (HO). We then used LASSO-selected PCs as the input for the final linear model.

The two-step selection process was successfully able to identify translatable PCs, as shown in Figure 3B. The heatmap shows the frequency of a given PC being selected across multiple rounds and cross validation folds of LASSO. Using a cut-off of 40 percent cumulative selection frequency, we identified 4 of the original 13 PCs are translatable. PC2_h1, PC5_h1, PC9_h1, and PC11_h1 (Figure 3B, highlighted in magenta), all had significant coefficients in the final linear model as well. Since LASSO can vary between different rounds of feature selection, Figure 3B illustrates representative results from a single round of feature selection. A more complete illustration of LASSO across ten rounds of feature selection is shown in Supplementary Figure 2F. This shows that the four translatable features that were ultimately selected are robust between different rounds of feature selection.

We visualized the predictions from the final linear model for each of mouse samples used (Figure 3C). The average, linear model predicted value for the samples increases between the WT, HET, and HO groups and qualitatively aligns with the true mouse sample categorical values. We do see that there is overlap in sample prediction values between the different groups and note that the homozygous TASTPM mice in particular exhibit larger within group variance compared to the WT and HET mice. Although this final linear model is not robustly predictive of disease model state, the interspecies model still has utility toward biological interpretation of cross-species translatable signatures.

Furthermore, we evaluated the final model and confirmed that the selected PCs were statistically robust and biologically meaningful using two evaluation procedures: size-matched models and phenotype permutation testing. Using leave-one-out cross validation (LOO CV), we calculated an RMSE value for the true translatable model built using PC2_h1, PC5_h1, PC9_h1, and PC11_h1. We then compared the true RMSE metric against null models. First, we generated size-matched models using random combinations of PCs and found our true model to be statistically significant (*p*-value < 0.014; Supplementary Figure 2E). This type of random test shows that the four PCs selected by the true model encode meaningful and unique information with regard to the disease outcome. Second, we generated permuted phenotype models for which we shuffled the original phenotype vector and repeated the full two-step feature selection process. We conducted a second type of null model building 100 times, and all 100 random runs failed at the LASSO feature selection step. With scrambled phenotype vectors, PCs were never selected frequently enough across runs and cross validation folds to satisfy the 40 percent selection threshold. Although we could not assign an empirical *p*-value to this second type of model, the result still meaningfully shows that the shape of the data is unique with regard to disease outcome. If many of the random phenotype models had low RMSE values, we would have to consider whether the shape of the data allows for easy prediction of categorical outcomes.

### Identification of Cross-Species Disease Signatures, Distinct From Aging

We identified four human PCs as encoding human disease relevant information also important for predicting mouse sample disease status. However, mice of four different ages were present in the dataset (2, 4, 8, and 18-months). The original mouse model study focused age-based evaluation on identifying transcriptomic signatures that were detectable early relative to histopathological phenotype and evaluating the temporal dynamics of transcriptomic signatures generally, and this age information was not explicitly accounted for in the TransComp-R framework alone (Matarin et al., 2015). As such, we utilized the age information in the mouse model dataset to evaluate the effects of age on the selected translatable components.

To determine whether the four translatable components identified thus far encoded disease-relevant information relative to age, we employed linear modeling (LM) (Figure 1, Step 3). We built a pair of linear models for each component: the first model was trained to predict mouse sample scores on the projected component using age alone as a factor, and the second model was trained to predict mouse sample score using both age and disease as factors. Comparing the two models allowed us to discern whether disease encoded information that was unique relative to differences resulting from sample age, resulting in a statistically significant improvement in the second model. AD is strongly associated with aging, and unlike in other disease areas where sample age could be treated as a confounding factor, modeling age-dependent effects is particularly important for AD. Thus, this modeling step is a relevant addition to the TransComp-R workflow in an AD context.

The results from LM analysis are shown in Figure 3D. Of the four translatable components identified based on mouse and human disease status, two components (PC9_h1 and PC11_h1) were also identified at a *p*-value threshold of 0.01 through LM analysis. PC9_h1 and PC11_h1 are considered to have disease-relevant information encoded in addition to information encoded by sample age. LM analysis does not preclude the importance of PC2_h1 and PC5_h1. Given the increased prevalence of human AD with age and its importance in disease progression, it is likely that there are disease relevant signatures that tightly associate with aging. We ultimately chose to focus on translatable components that encoded disease-relevant information separate from changes in mouse sample age for initial biological interpretation, as we suggested that these components would encode particularly robust cross-species disease signatures.

**FIGURE 4:**
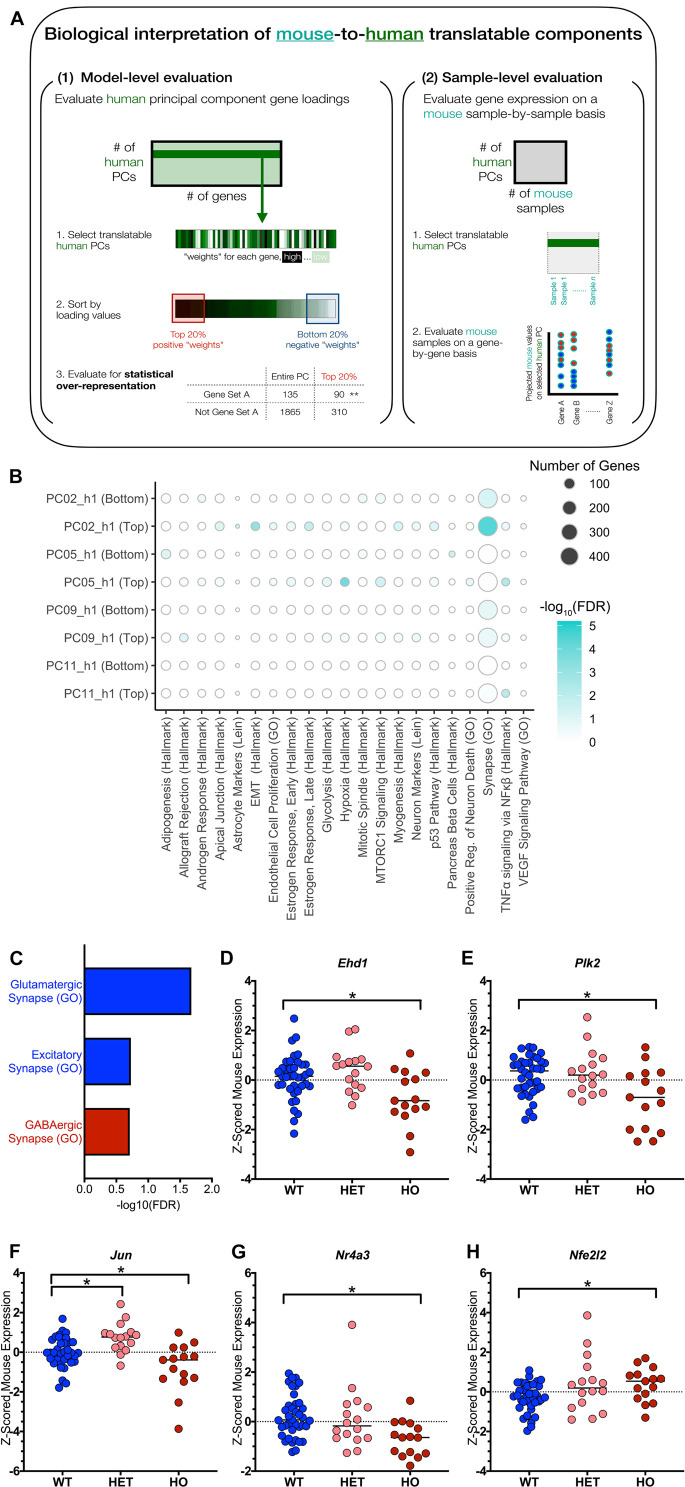
Biological interpretation of translatable signatures identifies excitatory synapse and TNFα signaling pathways. **(A)** Biological interpretation of TransComp-R modeling results spans model- and sample-level evaluation as outlined in the workflow shown. **(B)** Dot plot heatmap showing pathways that are statistically enriched at the top and bottom 20 percent of each principal component’s loadings relative to all the gene loadings for the PC. The full list of original pathways evaluated is in Supplementary Table 1. A pathway is included in the figure if it was enriched at an FDR < 0.20 in any of the eight groups (four PCs, top and bottom) evaluated. Dot size for pathway enrichment indicates the number of genes from the original gene set list that are also present in the PCA model and thus available for potential enrichment. **(C)** PC9_h1 is enriched for the Synapse (GO) pathway at both the top and the bottom of the PC. Conducting a secondary pathway enrichment analysis with synapse-specific pathways (full list in Supplementary Table 2) shows that glutamatergic and excitatory synapses are enriched at the bottom of the PC while GABAergic (inhibitory) synapses are enriched at the top of the PC. Negative log_10_ FDR values are shown for each pathway, and pathways are colored based on whether they were enriched at the top of PC9_h1 (red) or the bottom of PC9_h1 (blue). **(D–H)** TNFα signaling via NFκβ is enriched at the top of PC11_h1. Genes that contributed to the enrichment of the pathway were evaluated in terms of z-scored mouse gene expression. Of the 20 genes driving pathway enrichment, five genes had at least one significant *t*-test comparison between either the wild type (WT) and heterozygous TASTPM (HET) or WT and homozygous TASTPM (HO) samples (* adjusted *p*-value < 0.05; Benjamini-Hochberg corrected for multiple hypothesis testing). The median of each group is indicated in addition to the values for each sample.

Having identified and prioritized translatable components of interest, we next interpreted the biological signatures encoded in these components at a model-level and a sample-level (Figure 4A). The original PC space was generated only using the human data, and gene loading (loosely related to “weights”) interpretation could be viewed as a human AD-leaning interpretation (Figure 4A, left). As such, we also included sample-level evaluation that focused on mouse sample expression of genes that were important within the human model (Figure 4A, right).

Starting with model-level pathway analysis, we conducted statistical over-representation analysis comparing all genes in the model to genes found in the top 20 percent and bottom 20 percent of the genes for each PC. We looked for over-representation of gene set members at the top and bottom of the PC relative to the full list, and we started with a list of 68 broad pathways. The pathway list included MSigDB’s Hallmark gene sets, gene sets implicating specific CNS cell types, gene sets relating to the amyloid cascade in light of the TASTPM mouse model we analyzed, and gene sets representing both arms of the immune system (Supplementary Table 1). The methods section details this original list further, but an overarching goal was to start with broad, relatively non-overlapping categories that could guide further biological interpretation.

Figure 4B shows the results of this initial pathway enrichment analysis. We conducted pathway enrichment analysis for all four PCs identified through TransComp-R and first focused on PC9_h1 and PC11_h1, which were further identified through linear modeling. Focusing on PC9_h1 and PC11_h1, we saw that one of the neuron-oriented gene sets, Synapse (GO), was enriched at both the top and bottom of PC9_h1. On PC11_h1, TNFα signaling via NFκβ was enriched at the top of the PC.

### Excitatory Synapse Pathways Are Human Disease-Relevant and Classify TASTPM-vs-Control Mice

Focusing on PC9_h1, we found it curious that the Synapse (GO) gene set was enriched at both the top and the bottom of the gene set. We curated a second list of synapse-focused gene sets. We included gene sets for neurotransmitter-specific synapses as well as negative and positive regulation of synaptic processes, and the full list can be found in Supplementary Table 2. Conducting a second statistical over-representation analysis with this targeted list, we were able to understand that different categories of genes were driving enrichment of the broad Synapse (GO) category at the top and bottom of PC9_h1.

Glutamatergic (GO) and excitatory (GO) synapse gene sets were enriched at the bottom of PC9_h1 while the GABAergic (GO) synapse gene set was enriched at the top of PC9_h1 (Figure 4C). Looking at mouse samples projected onto PC9_h1, we determined that the top of PC9_h1 was associated with progressed disease in mice (Supplementary Figure 2A). There was minimal overlap between the gene set members driving the enrichment of the glutamatergic (GO) and excitatory (GO) synapse categories (five overlapping genes relative to 47 and nine enriched genes, respectively).

In the original study analyzing the human dataset used here, Berchtold et al. (2013) reported extensive changes in synapse-related genes in human AD patient samples, predominantly trending toward down-regulation. Specifically, glutamate and GABA receptor associated genes showed declining expression in both AD patients and older patients. Glutamate receptor trafficking genes were also affected. Multiple other subclasses of neurotransmitter receptors, not all of which were identified in our analysis, were also implicated.

The original mouse study identified synapse related changes as associating with plaque burden (Cummings et al., 2015; Matarin et al., 2015). Synapse-related changes at the gene level were detectable starting at 2 months and significant starting at 4 months. The authors utilized WGCNA to identify synaptic changes as a module of interest, and synaptic transmission, cell-cell signaling, and transmission of nerve impulses were subsequently identified pathways within the module. These changes were functionally validated via patch-clamp recordings of mouse hippocampal CA1 pyramidal neurons, and the original work noted glutamate release probability and spontaneous action-potential mediated activity were compromised.

Prior AD literature further confirms this biological inference. Synapse loss and loss of synaptic gene expression have been extensively correlated with cognitive impairment and AD progression (DeKosky and Scheff, 1990; Terry et al., 1991; Scheff and Price, 2003; Spires et al., 2005). Focusing on excitatory synapses specifically, there exists excitotoxicity theories positing that aberrant and excessive synapse excitation via glutamate and N-methyl-D-aspartate (NMDA) receptors results in localized neurotoxicity and could serve as an initiator in early AD progression (Esposito et al., 2013). The interplay and known causalities of excitotoxicity and the amyloid cascade have been reviewed in the literature (Hynd et al., 2004). Furthermore, Memantine is an existing FDA-approved therapy that works as a non-competitive NMDA antagonist for AD (Esposito et al., 2013).

Looking at the original human study, the original mouse study, and the AD literature broadly, we were able to conclude the following about our biological inference on synapses. This signature was strongly present in both the original human and original mouse studies, suggesting it is a signature that could have been identified via observational comparison of the datasets. This signature is also known to be AD associated and has been considerably explored in research and therapeutic development. While the identification of this signature via observational comparison does not showcase the unique utility of TransComp-R for cross-species analysis, we did consider identification of well-known AD signatures to be positive validation of TransComp-R as a methodology. Lastly, we note that enrichment of these signatures here is driven by synergistic gene expression in the human and mouse datasets, and the mathematically identified glutamatergic and excitatory synapse signatures directionally align with functional changes in AD reported in the literature.

### TNFα Signaling via NFκβ Is a Cross-Species Signature Potentially Confounded by Mouse Model Genetics

Focusing on PC11_h1 identified at the intersection of TransComp-R and LM analysis, we saw that TNFα signaling via NFκβ was enriched on PC11_h1. Since statistical over-representation analysis focuses on the human data-based model, we followed up by looking at the mouse sample expression of genes driving enrichment of the inflammatory cytokine signature. We first identified all 20 genes that were members of the TNFα signaling via NFκβ gene set and had loadings in the top 20 percent of PC11_h1. We then looked at mouse sample gene expression for each of these genes. Conducting two-way *t*-tests between WT, HET, and HO mice, we highlighted all genes that had at least one intergroup, significant comparison relative to WT samples in Figures 4D–H. These five genes could be considered mouse-to-human synergistically identified genes as they were important in the human PCA model and had statistically significant differences in expression in mouse based on disease status.

In the original human cohort study, inflammatory innate immune response was identified as a signature associated with disease (Cribbs et al., 2012). It is worth noting that these signatures were more pronounced in general aging as compared to AD. Focusing specifically on members of the TNFα signaling via NFκβ gene set, TNFα specifically was not detected in the original microarray dataset due to sensitivity but measured as being increased, albeit not significantly, in both AD and aging through qPCR. In the original mouse study, immune response as a general category had increased hippocampal expression in the 8 and 18-month TASTPM samples. TNFα signaling via NFκβ was not explicitly identified.

TNFα signaling via NFκβ is a cytokine-initiated inflammatory signaling cascade that is well documented in the AD literature (Akiyama et al., 2000; Cheng et al., 2014). Interestingly, multiple of the five genes identified via synergistic mouse-to-human expression are also shown in the literature to have direct interactions with the amyloid cascade. *EHD1* encodes an ATP- and membrane-binding protein (Figure 4D). EHD1 and other members of the EHD protein family are necessary for unidirectional dendritic transport, affect axonal transport, and modulate levels of the enzyme beta secretase (BACE1) in both compartments (Buggia-Prévot et al., 2013). *PLK2* encodes the serine/threonine protein kinase PLK2 (Figure 4E). PLK2 has been reported to phosphorylate amyloid precursor protein (APP) *in vitro* (Lee et al., 2017) and reduce plaque levels upon inhibition *in vivo* in mouse (Lee et al., 2019).

In the datasets used for this case study, there was decreased *EHD1* and *PLK2* expression in AD patients relative to controls and decreased *Ehd1* and *Plk2* expression in homozygous TASTPM mice relative to wild-type mice. Interestingly, *EHD1* shows increases in AD patients relative to non-cognitively impaired controls across different cortical regions in multiple human studies aggregated through the Alzheimer DataLENS project (Bihlmeyer et al., 2019). *PLK2* showed decreased expression across multiple brain regions within the same database.

TNFα signaling in general has been reported to directly affect APP processing so perhaps the interactions of our mouse-to-human synergistic genes with the amyloid cascade is unsurprising (He et al., 2007; Cheng et al., 2014). Nonetheless, it is important to underscore previously reported ties between the specific genes we highlight and APP processing. Given that the TASTPM mouse is driven by mutations in the amyloid cascade and that numerical significance in the TransComp-R workflow is derived from a signature’s importance across both species, we speculate that statistical significance of this result could be confounded by TASTPM mouse model genetics despite the signature’s validated biological significance in both AD mouse models and human AD. Additionally, we note some differences in directionality of gene expression changes between the datasets used in this TransComp-R case study and those in the broader literature. This could be explained in part by the different brain regions being profiled but globally does suggest further limitations with regard to the genes contributing to this signature’s mathematical enrichment.

### Identification in a Second Independent Cohort of Human Pathways Important for TASTPM Mouse Outcomes

In the first case study, we combined a single mouse and single human dataset, and we demonstrated that TransComp-R can successfully identify cross-species signatures using a permissive list of human DEGs as input. The biological inferences included a well-established excitatory synapse signature and an inflammatory cytokine signature that is documented in the AD literature but could have been numerically confounded by mouse mechanisms in this particular study. Taking these lessons from the first study, we constructed a second case study using the same mouse model samples and incorporating the second human cohort that was discussed during PCA model construction (Figures 2C,D). This second human cohort included more granularity in terms of patient outcomes, and we hoped to incorporate the increased clinical information into the case study workflow for biological inference. A second goal of conducting TransComp-R with a second, independent human cohort was to assess the reproducibility and robustness of the TransComp-R pipeline for cross-species analysis using a broader list of input genes.

**FIGURE 5:**
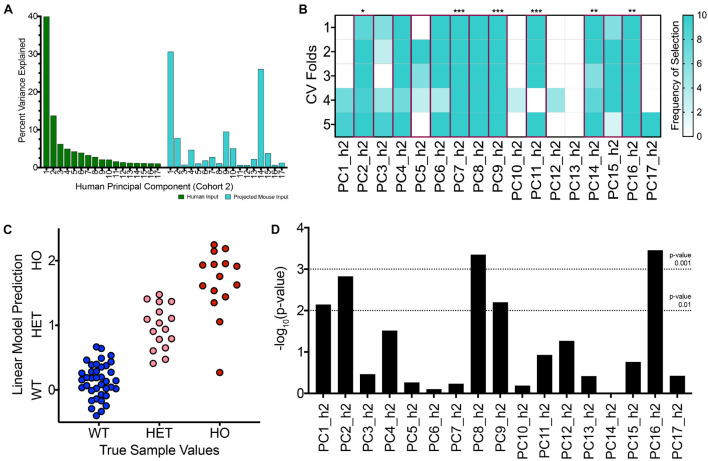
With a second, independent human cohort, TransComp-R modeling successfully identifies translatable, human principal components that are important for TASTPM mouse sample outcomes. **(A)** Percent variance explained by each human PC for the original human dataset used to generate the PCA model (GSE1297, cohort 2; green) and the projected TASTPM mouse dataset (turquoise). **(B)** Frequency of human PC (PC_h2) selection across ten rounds of 5-fold cross validation using LASSO feature selection. PCs selected at the selection frequency threshold of 40 percent are highlighted in magenta with the significance of the linear model coefficient weight provided (* < 0.05, ** < 0.01, *** < 0.001). **(C)** Visualizing the linear model predictions on a mouse sample-by-sample basis from the final translatable model across the wild type (WT), heterozygous TASTPM (HET), and homozygous TASTPM (HO) groups. **(D)** Results from linear modeling evaluating which PCs contain information that is relevant for disease-status, in addition to the information encoded by sample age.

To initiate a new TransComp-R case study, we generated a PCA model for the new human cohort where there was weaker categorical disease separation between patients on PC1_h2 compared to the first case study described (Figures 2C,D). We next projected the same TASTPM and wild type control mouse samples into the new human PCA space. Mathematically, this projection involved combining mouse gene expression with the ‘weights’ (loadings) calculated using the second human cohort. The variance accounted for by mouse sample scores on each PC within the projected mouse matrix is shown in Figure 5A. The percent variance of the human dataset in the original human PCA model is also shown. We see monotonic decrease in percent variance explained for the human data with increasing PC number, as inherent to PCA. The projected mouse data do not follow this monotonic trend. We see that PC3_h2 explained less percent variance of the projected variance relative to both PC2_h2 and PC4_h2. We were also surprised to see PC9_h2 and PC14_h2 show a strong jump in percent variance explained for the projected mouse data matrix.

Having projected the mouse samples for concomitant cross-species analysis, we proceeded to identify translatable components. We conducted LASSO feature selection and linear modeling using the mouse samples projected into human PC space and the mouse sample outcomes. The results of this feature selection process are shown in Figure 5B. LASSO feature selection selected a greater number of features compared to the first case study. Given the greater number of input features to the linear model, not all PCs selected via LASSO were statistically significant in the final translatable model. Additional results from LASSO are shown in Supplementary Figure 3.

We visualized the predicted value for each mouse sample using the 11-component linear model, and the sample predictions are shown in Figure 5C. Similar to the first case study, the average predicted value for samples increases from WT to HET to HO groups and qualitatively aligns with the true mouse sample categorical values initially assigned. There is overlap in sample predictions between the groups. The homozygous TASTPM mice again exhibit the greater within group variance, and the homozygous TASTPM sample predicted to be similar to WT samples was from the 2-month time point (Supplementary Figure 3G).

As a result, we followed up with linear modeling to identify translatable components that encoded disease-relevant information separate from age. Multiple PCs encoded information that was relevant for delineating samples based on disease status in a way that was separate from sample age. Specifically, PC1_h2, PC2_h2, PC8_h2, PC9_h2, and PC16_h2 had *p*-values less than 0.01 from linear modeling (Figure 5D).

There were three translatable components that were selected via LASSO feature selection, statistically significant in the mouse-to-human translatable model, and explained disease-relevant information distinct from aging in a separate linear model: PC2_h2, PC9_h2, and PC16_h2. Mouse samples are visualized along these three human PCs in Supplementary Figures 3A,B.

We compared the true linear model against null models. For the first of two null model comparisons, we compared the LOO RMSE value for the true model against null, random PC models. Our true model had an empirical *p*-value less than 0.067 (Supplementary Figure 3E). For the second of two null model comparisons, we sought to compare the LOO RMSE value for the true model against null, random phenotype models. Of 100 null model initializations, no null models succeeded in selecting any components at the LASSO step.

### Complement and TAMR Agonist Signatures Identified From LOAD Human Patient and TASTPM Mouse Data

**FIGURE 6:**
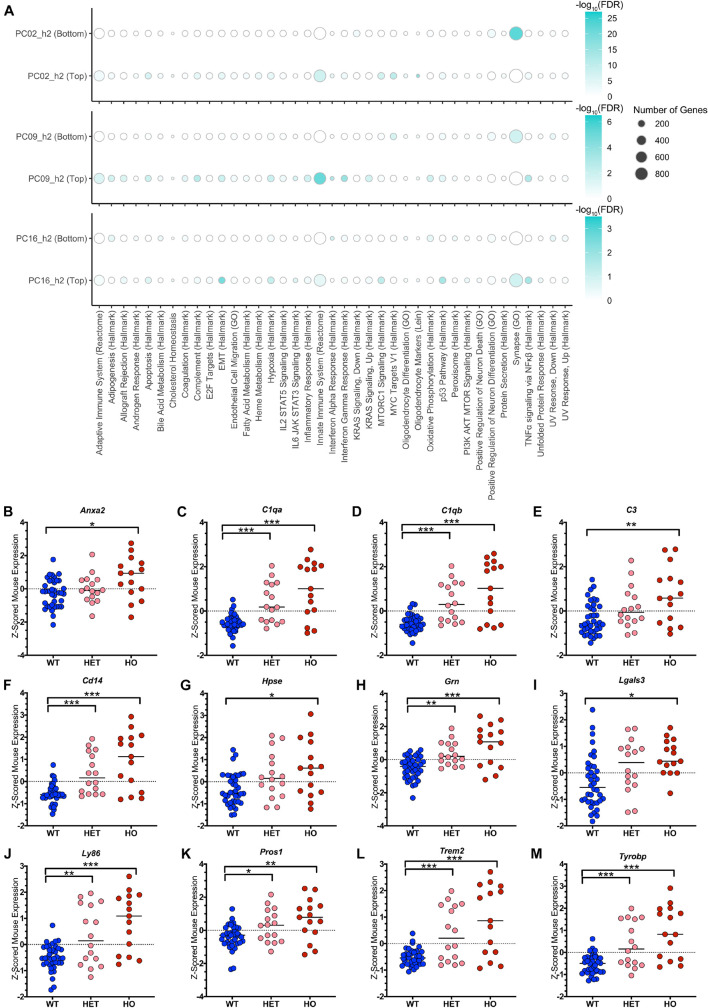
TransComp-R identifies complement and TAM receptor agonist signatures from LOAD human patient and TASTPM mouse samples. **(A)** Dot plot heatmap showing pathways that are statistically enriched at the top and bottom 20 percent of each principal component’s loadings relative to all genes present in the PC. The full list of original pathways evaluated in Supplementary Table 1. A pathway is included in the figure if it was enriched at an FDR < 0⋅05 in any of the six groups (three PCs, top and bottom) evaluated. Dot size for pathway enrichment indicates the number of genes from the original gene set list that are also present in the PCA model and thus available for potential enrichment. Color intensity represents the -log_10_(FDR) value and is normalized to the maximum -log_10_(FDR) value on a PC-by-PC basis. **(B–M)** The Innate Immune System (Reactome) pathway is enriched at the top of PC9_h2. Genes that contributed to the enrichment of the pathway were evaluated in terms of z-scored mouse gene expression. Genes that had a significant *t*-test comparison between either the wild type (WT) and heterozygous TASTPM (HET) or WT and homozygous TASTPM (HO) samples and were associated with a secreted protein in the UniProt database are shown. (* < 0.05, ** < 0.01, *** < 0.001, Benjamini-Hochberg corrected for multiple hypothesis testing; the median of each group is indicated in addition to the values for each sample).

Turning toward biological interpretation of the models, we conducted pathway analysis on the three translatable components that were identified separately through both TransComp-R and linear modeling delineating aging and disease progression. Innate immune system activation was strongly enriched at the top of both PC2_h2 and PC9_h2, associating with disease (Figure 6A).

On PC9_h2, there was a robust, coordinated enrichment of multiple immune-related pathways. Specifically, the most numerically significant pathways enriched at the top of the PC were the Innate Immune System (Reactome), Interferon Gamma Response (Hallmark), Interferon Alpha Response (Hallmark), TNFα signaling via NFκβ (Hallmark), and Complement (Hallmark) pathways. The same immune pathways were enriched on PC2_h2, and we see mouse sample score similarity along PC2_h2 and PC9_h2 as compared to PC16_h2 (Supplementary Figures 3A,B). On PC2_h2, there was also enrichment of oligodendrocyte pathways (e.g., Oligodendrocyte Differentiation (GO)) and the Adaptive Immune System (Reactome) alongside innate immune system pathways. Full pathway enrichment information for all three translatable components in available in Supplementary Table 3. Given the concurrent enrichment of a unified block of innate immune pathways on PC9_h2, we chose to focus on PC9_h2 for initial biological analysis of cross-species disease inferences.

It is worth noting that pathway enrichment is a model-level analysis, which is human-leaning. As a result, we introduced sample-level analysis to identify pathway member genes that were important in mouse as well. 196 genes contributed to the enrichment of the Innate Immune System signature on human PC9_h2, and 46 of these genes had statistically significant differences in expression in mouse based on disease status. 25 of the 46 genes were also significant via the same analysis on PC2_h2.

We were interested in evaluating genes that could aid in hypothesis generation for potential therapeutics. We utilized the Universal Protein Resource (UniProt) database to identify genes with an associated, secreted protein, reasoning that receptor-ligand interactions could be more readily targeted via therapy. Evaluating the list of synergistic mouse-and-human genes using the criteria fully described in the Methods, we identified 12 genes of interest. Mouse gene expression for these genes is shown in Figures 6B–M. Three genes identified are components of the complement cascade (*C1qa, C1qb, C3*; Figures 6C–E), in alignment with enrichment of the Complement (Hallmark) pathway. The LOAD-relevant genes *Trem2* and *Tyrobp* were also significant (Figures 6L,M). Lastly, we identified two ligands for the Tyro3 / Axl / MerTK (TAM) receptor family: Galectin-3 (*Lgals3*) and Protein S (*Pros1*) (Figures 6I,K).

Classical complement activation was enriched among up-regulated AD-associated genes in the original human studies, although in Cribbs et al. (2012), complement and other innate immune signatures were much more strongly associated with normal aging as compared to AD. In the oldest homozygous TASTPM mice and oldest tau mice in the original mouse model study, immune system involvement was a shared signature. WGCNA identified multiple hub genes – *C1aq*, *C1qb*, *C1qg*, *Fcer1g*, *Hcph*, and *Trem2* – multiple of which were identified in our study (Matarin et al., 2015).

Turning from observational comparison to the broader AD literature, we find validation of our complement cascade and TYROBP inferences. The complement cascade has been reported to mediate microglial pruning of neuronal synapses in development and behaves aberrantly in neurodegenerative disease (Schafer et al., 2012; Hong and Stevens, 2016; Hong et al., 2016). Specific to genes enriched in our analysis, elevation of C1q has been reported to precede plaque deposition in AD mouse models. While inhibition of C1q or other members of the complement cascade attenuates synapse loss, it is not yet clear if initial synaptic pruning is a neuroprotective or aberrant response (Hong et al., 2016; Bartels et al., 2020).

TYROBP is an adapter protein that forms a signaling complex with TREM2, which has gained significant attention stemming from the identification of a rare, risk-associated variant of TREM2 through genome wide association studies (Gratuze et al., 2018). TYROBP itself has been reported to be upregulated in LOAD patient brains at the transcript level across multiple brain regions (Zhang et al., 2013). In a previous integrative computational analysis of human brain tissue gene expression, innate immune and microglial processes were identified as the most important. TYROBP was identified as a potential causal regulator through Bayesian inference within the module (Zhang et al., 2013). Multiple other studies have reported *TYROBP* and *TREM2* to occupy ‘hub gene’ roles (Holtman et al., 2015; Matarin et al., 2015) and single cell transcriptomic profiling of microglia identified a TREM2-dependent step in the transition of microglia to a disease associated state (Keren-Shaul et al., 2017).

We noted that two of the 12 genes encoded agonists for the TAM family of receptors (Figures 6I,K). Specifically, Protein S is a ligand for Mer and Tyro3, and Galectin-3 has thus far been reported to interact with Mer (Pierce and Keating, 2014). Interestingly, many members of the broader TAM family were polarized along translatable component PC9_h2. We examined the PCA model loadings for all TAM family receptors and ligands. We found that Tubby (*TUB*), Galectin-3 (*LGALS3*), Axl (*AXL*), and Protein S (*PROS1*) all had strong positive loadings on PC9_h2 associating with disease (ranked loadings in the second, third, ninth, and tenth percentile, respectively). Conversely, Tyro3 (*TYRO3*) and Mer (*MERTK)* had strong negative loadings that associated with control samples along the same translatable component (88th and 89th loading percentile, respectively). Gas6 (*GAS6*) and Tubby-like Protein 1 (*TULP1*) were not strongly ranked along PC9_h2 in the model.

The TAM receptor family has been reported to play a role in regulating microglial phagocytosis (Fourgeaud et al., 2016; Butovsky and Weiner, 2018; Burstyn-Cohen and Hochberg, 2021; Huang et al., 2021). Canonical TAMR ligands such as Gas6 and Protein S bind TAM receptors through the C-terminal region and phosphatidylserine via the N-terminal region, bridging TAM expressing cells with phagocytic targets such as apoptotic bodies (Lemke and Rothlin, 2008). Protein S is frequently described as a ligand for the TAMR family, while Galectin-3 interactions with the TAMR family have been lesser characterized. Galectin-3 was identified to bind Mer via functional cloning and co-immunoprecipitation, and Galectin-3 treatment resulted in Mer auto-phosphorylation *in vitro* (Caberoy et al., 2012). Subsequently, Galectin-3 has been shown to opsonize cells and modulate mouse microglial phagocytosis involving Mer (Nomura et al., 2017).

Protein S and Galectin-3 both have other known functions outside of the TAMR family. Protein S has functions in the coagulation and complement cascades, and Protein S knockout in mice results in embryonic lethality (Burstyn-Cohen et al., 2009). In addition to interacting with Mer, Galectin-3 has also been shown to bind TREM2 *in vitro* and activate TLR4 (Thomas and Pasquini, 2018; Boza-Serrano et al., 2019; Puigdellívol et al., 2020). In cell line and mouse model contexts, microglia have been shown to increase Galectin-3 expression in response to inflammatory stimuli and when plaque-associated (Nomura et al., 2017; Boza-Serrano et al., 2019; Puigdellívol et al., 2020). Galectin-3 knockout (Gal3KO) also has varied impacts on 5XFAD mouse outcomes. Specifically, 5XFAD/Gal3KO mice showed decreased plaque burden, plaque perimeter, and soluble Aβ40 compared to 5XFAD mice while soluble Aβ42 increased relative to 5XFAD mice, all with age specificity (Boza-Serrano et al., 2019).

Transitioning to omics studies, *Axl* and *Lgals3bp* were identified as upregulated in the late-stage, disease-associated microglial cluster from CK-p25 mouse single cell RNA-sequencing (Mathys et al., 2017). *Axl* was identified as being upregulated in late stage disease associated microglia in single cell RNA-sequencing of 5XFAD mice (Keren-Shaul et al., 2017). Proteomic comparison of homeostatic and amoeboid-phagocytic mice microglia identified increased Galectin-3 in phagocytic microglia (Krasemann et al., 2017). Protein S was also identified in proteomic evaluation of 5XFAD mouse hippocampi and increase was subsequently validated in human AD patient serum (Kim et al., 2019). In human studies, Galectin-3 has been found at the protein level to be increased in AD patients relative to controls in serum profiling as well as in the cortex (Wang et al., 2015; Tao et al., 2020).

Currently, mechanistic roles of the TAM family of receptors and ligands are lesser studied in AD. Mer and Axl specifically have been implicated in regulating phagocytic functions in microglia, and, specific to AD, Mer, Axl, and TREM2 co-expression has been observed on activated macrophages near plaques (Savage et al., 2015; Fourgeaud et al., 2016). Interestingly, we found a recent study investigating TAM family expression at the mRNA level in human patients in relation to TLR signaling. Herrera-Rivero et al. (2019) reported that TAM receptor levels were relatively unchanged in the frontal cortex across different stages of AD but also identified Protein S and Galectin-3 as ligands of interest. Specifically, Protein S and Galectin-3 were increased at the mRNA level in moderate but not late stage AD patients, and the two ligands showed similar expression profiles across different stages of disease (control, incipient, moderate, severe AD). The authors proceeded to validate their findings with a focus on TLR signaling via combination ligand treatments in Thp-1 culture. Huang et al. (2021) recently evaluated the role of TAM receptors Axl and Mer in an *APP/PS1* mouse model and postmortem human brain tissue. The authors identified Gas6 coating of amyloid plaques and upregulation of Axl at the protein level in plaque-adjacent microglia with consistent Mer. Crossing Axl and Mer knockout mice with *APP/PS1* mice, the authors determined that Mer was particularly critical for facilitating phagocytic functions in microglia while both Axl and Mer were important for sensing and migrating to amyloid plaques. The unique roles of Axl and Mer in microglial response is intriguing, given that both TAMR agonists identified in our study bind Mer but not Axl.

All mouse-to-human biological inferences and associated literature validation are summarized in Table 1.

**TABLE 1:** Biological inference summary from mouse-to-human cross-species analysis.

Biological Inference	References
**Excitatory and Glutamatergic synapse** enrichment anti-correlates with disease	DeKosky and Scheff, 1990; Terry et al., 1991; Scheff and Price, 2003; Hynd et al., 2004; Berchtold et al., 2013; Esposito et al., 2013; Matarin et al., 2015
**TNFα signaling via NFκβ** is a cross-species translatable signature that contributes to separation of control and disease mice	Akiyama et al., 2000; Cribbs et al., 2012; Cheng et al., 2014
**Microglial Activity** • Innate immune activity is a cross-species translatable signature. • *TYROBP, TREM2*, and complement cascade genes (*C1QA, C1QB, C3)* contribute to the human disease signature and are significantly increased in TASTPM heterozygous and homozygous mice	Akiyama et al., 2000; Cribbs et al., 2012; Schafer et al., 2012; Zhang et al., 2013; Holtman et al., 2015; Matarin et al., 2015; Hong and Stevens, 2016; Hong et al., 2016; Keren-Shaul et al., 2017; Gratuze et al., 2018; Salih et al., 2019; Bartels et al., 2020
**TAM family receptor agonists** (Protein S and Galectin-3) associate with disease	Pierce and Keating, 2014; Savage et al., 2015; Wang et al., 2015; Fourgeaud et al., 2016; Keren-Shaul et al., 2017; Krasemann et al., 2017; Mathys et al., 2017; Butovsky and Weiner, 2018; Boza-Serrano et al., 2019; Herrera-Rivero et al., 2019; Kim et al., 2019; Puigdellívol et al., 2020; Tao et al., 2020; Burstyn-Cohen and Hochberg, 2021; Huang et al., 2021

## Discussion

In this study, we applied a novel computational framework, TransComp-R and mixed linear modeling, to concomitantly analyze human and mouse AD transcriptomics and identify translationally relevant signatures, distinct from changes in age. An advantage of this framework is the capability to obtain insights beyond the typical observational comparison of human versus mouse datasets which can only find apparent commonalities. At the same time, our method also ascertains signatures found in the traditional observational approach, as should be expected: signatures strongly enriched in each dataset separately can readily exhibit strong numeric synergy in the concomitant analysis. Our approach thus represents a significant advance beyond previous cross-species analyses in the field. Typically, such studies have considered animal and human data sequentially and with focus on directly observed commonalities. That is, the mouse model and human datasets are analyzed separately and sometimes using different methodologies, with differential expression and pathway enrichment results then compared in a Venn Diagram-like fashion to identify what is clearly shared between both species.

We identified multiple mouse-to-human translatable signatures that found validation in the literature. Excitatory synapse signatures were identified via observational comparison between the original human and mouse studies, further validated in older AD literature, and have been targeted via existing AD therapies (DeKosky and Scheff, 1990; Terry et al., 1991; Scheff and Price, 2003; Hynd et al., 2004; Spires et al., 2005; Berchtold et al., 2013; Esposito et al., 2013; Cummings et al., 2015; Matarin et al., 2015). TNFα signaling via NFκβ is a signature that was validated in literature observing cytokine-mediated inflammation as a component of AD progression (Akiyama et al., 2000; Cheng et al., 2014). It is possible that TASTPM mouse model genetics confounded the numeric significance of this finding, and in response, we noted the need to consider both the mathematical model and the biology when interpreting TransComp-R outcomes (He et al., 2007; Buggia-Prévot et al., 2013; Lee et al., 2017, 2019).

Complement cascade and TREM2/TYROBP inferences from our study were validated in integrative computational AD literature and in literature oriented toward LOAD (Cribbs et al., 2012; Schafer et al., 2012; Zhang et al., 2013; Hong and Stevens, 2016; Hong et al., 2016; Keren-Shaul et al., 2017; Gratuze et al., 2018; Bartels et al., 2020). The ability of TransComp-R to identify well-known hub genes, such as TREM2 and TYROBP, identified through other computational methods provided a positive confirmation of our modeling methodology, especially given analysis using a traditional amyloid mouse model dataset. TREM2 and TYROBP are not directly modulated by TASTPM transgenes, and identification of this AD-relevant signature in TransComp-R analysis suggests that the TASTPM model embodies these translational mechanisms. This suggests the TASTPM mouse model could be suitable as a preclinical model targeting associated microglial pathways. We suggest that TransComp-R could be useful for rational selection of mouse models in this way – the TransComp-R case study workflow we present in this study could be expanded to multiple AD mouse models. We could envision conducting cross-species analyses with expanded coverage of multiple mouse models to identify those that demonstrate robust translational enrichment of pathways relevant for a therapeutic mechanism of action. Rational selection could entail selecting several mouse models driven by different transgenes but sharing therapeutic target-relevant dysregulation for preclinical evaluation of therapies. For example, previous literature demonstrating increased TREM2 expression in other amyloid mouse models suggests that evaluating additional amyloid mouse models with a TREM2 focus could be a potential next step (Ulrich et al., 2017; Karanfilian et al., 2020).

Lastly, we identified purported agonists (Protein S and Galectin-3) of the TAM receptor family through cross-species analysis. Previous literature has described the TAM receptor family, especially the receptor Axl and Galectin-3/Galectin-3 binding protein, as being upregulated in disease and in disease-associated microglia (Holtman et al., 2015; Krasemann et al., 2017; Yin et al., 2017; Butovsky and Weiner, 2018; Boza-Serrano et al., 2019; Puigdellívol et al., 2020). However, mechanistic roles for TAM family receptors and agonists have been lesser studied in the AD literature. In a recent study, Huang et al. (2021) generated crosses between *APP/PS1* and *Axl-/-, Mertk-/-* knockout mice. The authors demonstrated that both Axl and Mer are important for microglial sensing and migration toward amyloid plaques while Mer is particularly important in facilitating microglial phagocytosis of and processing of plaques into dense-core form. In our study, Protein S and Galectin-3, two TAMR agonists that have not been shown bind Axl, are significantly disease associated while TAM receptors Tyro3 and Mer were strongly control associated along the same translatable principal component. We hypothesize that TAMR ligands with greater affinities to Mer could alter the relative and absolute expression of the three TAM receptors on microglia to preferentially push microglia into a therapeutically beneficial plaque-sensing, non-inflammatory, and competent-phagocyte state. It is worth noting that Protein S and Galectin-3 have other known functions apart from the TAMR family, such as Protein S’s interaction with members of the coagulation and complement cascades. Thus, future work is required to evaluate the mechanistic relationship between Protein S, Galectin-3, and the TAM receptor family in the context of microglial state and function.

We focused our case study framework on individual datasets that were matched by brain region. This study-by-study analysis framework, as opposed to aggregating datasets, could facilitate rational selection of specific mouse models in a mechanism-dependent fashion, as described immediately above. However, TransComp-R does not preclude comparisons across datasets with further differentiating factors. Presuming effective aggregation and normalization of data, the initial human or initial mouse matrix could combine data from multiple studies to increase sample number and heterogeneity captured.

Furthermore, TransComp-R is methodologically amenable to data types beyond the microarray datasets leveraged in this study. Comparing different transcriptomic data types, RNA-sequencing data has a broader dynamic range compared to microarray data. As such, we expect that the findings in this study represent a conservative set of cross-species results. We hypothesize that many of our findings could be validated in RNA-sequencing datasets and that additional signatures beyond the limit of detection for microarray data could be inferred using RNA-sequencing datasets. Additionally, the initial TransComp-R study (Brubaker et al., 2020) involved concomitant analysis of proteomic and transcriptomic data. This study demonstrated that TransComp-R is effective for other omics data not highlighted in our study, as the computational workflow does not mathematically preclude its application to various data types. Furthermore, Brubaker et al. (2020) demonstrated that TransComp-R can be applied across omics platforms and methodologies. In the case of cross-data type analysis, we can envision that that differences in data type such as coverage and dynamic range could affect the types of genes and pathways amenable to enrichment.

The mouse dataset utilized in this study included heterozygous and homozygous TASTPM samples. We encoded the disease status of the mouse samples in a linearly increasing fashion between wild-type, heterozygous, and homozygous mice. The transcriptomic data need not differ in a linear fashion, especially between the heterozygous and homozygous disease mice, and encoding disease status in this fashion could favor specific types of mRNA expression profiles for TransComp-R selection. There are alternate modeling strategies that could be employed. For example, we could employ one hot encoding to differentiate the three disease categories in a more independent fashion. We could also envision assigning mouse samples a continuous disease score based on sample-specific histopathological burden or behavioral examination scores. We could also envision combining the heterozygous and homozygous mice into a single category. Each of these three alternate strategies would preferentially identify genes that differentiate disease and control mice in a different, nuanced way as compared to this study. Additionally, TransComp-R could be applied with more granular disease outcomes such as a behavioral test score in mice or a cognitive exam score for human patients.

TransComp-R is a directional workflow that begins with constructing a PCA space. In this study, we first constructed a human PCA space using a permissive list of differentially expressed genes based on binary disease status and then constructed a separate human PCA space with an exploratory list of human-to-mouse homologous genes. Were our interest in a specific clinical outcome, we could envision generating a list of DEGs specific to the outcome (i.e., genes selected by regressing against Mini-Mental State Examination scores over time or against NFT score in postmortem histopathology) for a more supervised initial PCA space.

In summary, this analysis provides proof-of-concept for the utility of TransComp-R in translational analysis of mouse and human AD datasets. By integrating mouse and human datasets within the same analysis framework, we have shown that TransComp-R can identify important cross-species signatures that do not necessarily dominate in at least one of the datasets separately. We identified multiple biological inferences that were present at the signature level in human postmortem data and important in the context of mouse model disease status, including insights validated by previous literature as well as lesser studied insights worth further dedicated investigation, specifically the enrichment of TAM receptor family agonists. Furthermore, we were able to identify human AD-relevant signatures using existing, publicly available mouse model data from a traditional AD mouse model. Future work applying this case study framework to multiple AD mouse models could guide rational selection of models of preclinical evaluation of therapies.

## Data Availability Statement

Publicly available datasets were analyzed in this study. All gene expression datasets analyzed in this study were previously published and publicly available through Gene Expression Omnibus. They are available with the following specific datasets accession numbers were GSE1297 (human), GSE48350 (human), and GSE64398 (mouse). The analysis code for this work is available in a Github at https://github.com/meejlee/ADCrossSpeciesAnalysis.

## Author Contributions

ML, DB, and DL designed the study in association with BH. ML conducted publicly available data curation, formal analysis, and visualization. ML, MC, and CW contributed to methodology development. ML and CW wrote analysis software. CW contributed to software validation. ML and DL wrote the manuscript. All authors contributed to the article and approved the submitted version.

## Conflict of Interest

The authors declare that the research was conducted in the absence of any commercial or financial relationships that could be construed as a potential conflict of interest.

## Publisher’s Note

All claims expressed in this article are solely those of the authors and do not necessarily represent those of their affiliated organizations, or those of the publisher, the editors and the reviewers. Any product that may be evaluated in this article, or claim that may be made by its manufacturer, is not guaranteed or endorsed by the publisher.

## Footnotes

1
https://www.mathworks.com/matlabcentral/fileexchange/77987-transcompr
