# Supplementary material for: Computational Interspecies Translation Between Alzheimer’s Disease Mouse Models and Human Subjects Identifies Innate Immune Complement, TYROBP, and TAM Receptor Agonist Signatures, Distinct From Influences of Aging
Source: Front Neurosci. 2021 Sep 30;15:727784. doi: 10.3389/fnins.2021.727784 (PMC8515135; doi:10.3389/fnins.2021.727784)
Supplement: Supplementary file 1 [file Data_Sheet_1.docx]

Supplementary Material

**Supplementary Figures**
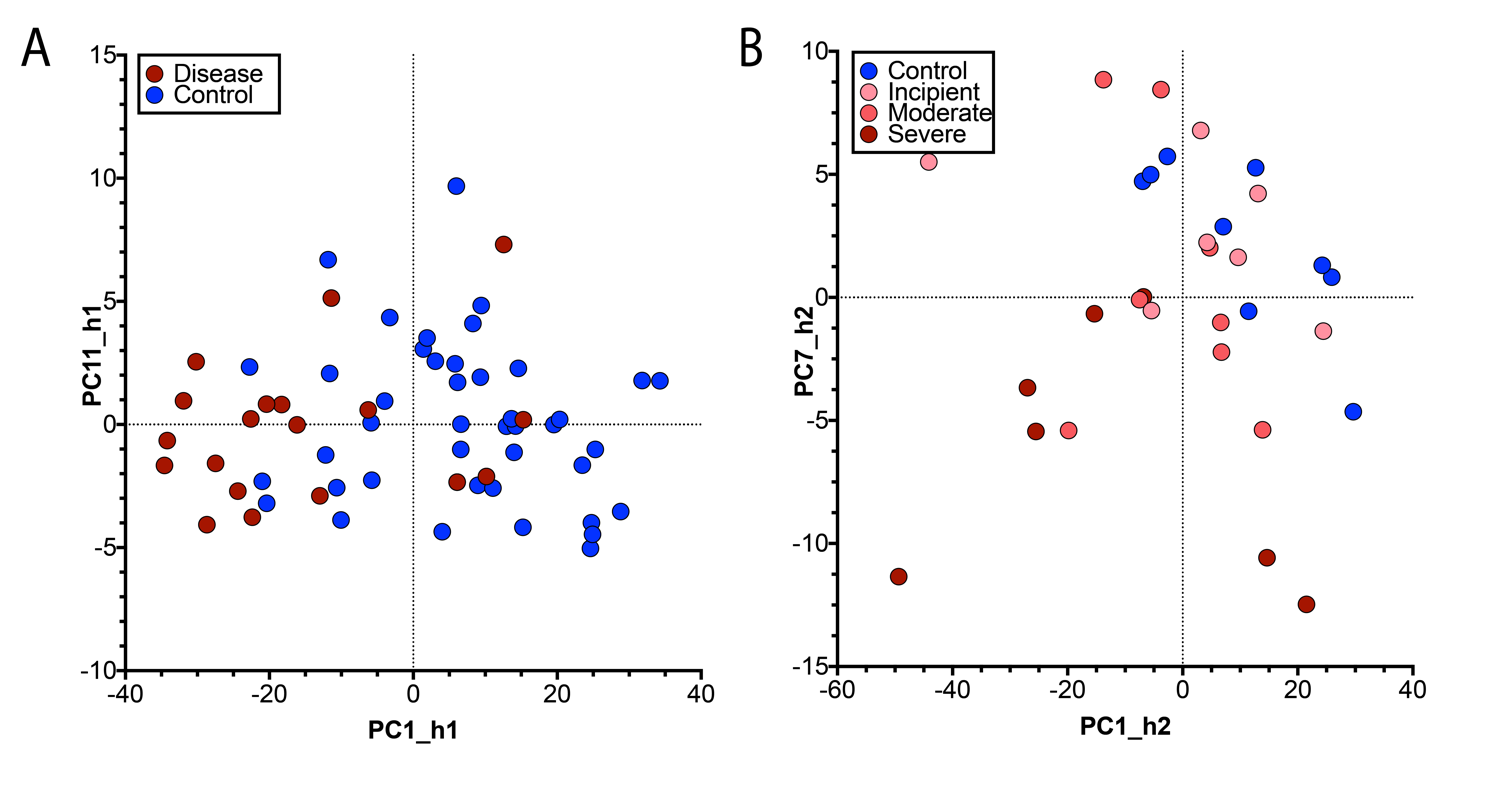


**Supplementary Figure 1. Additional evaluation of original human datasets**

(A) Principal component biplot showing 62 hippocampal human samples (GSE48350, human cohort 1) in PC1_h1, PC11_h1 space. The original, feature-level input to the PCA was genes differentially expressed between control (blue) and disease (red) patients at a permissive threshold of Benjamini Hochberg-adjusted p-value < 0.20 with no fold change criteria. PC1_h1 and PC11_h1 were identified as associated with disease-related variance based on linear regression in Figure 2c.

(B) Principal component biplot showing 31 hippocampal human samples (GSE1297, human cohort 2) in PC1_h2, PC7_h2 space. The original, feature-level input to the PCA was all genes with human-to-mouse homologs. PC1_h2 and PC7_h2 were identified as associated with categorical disease status based on linear regression in Figure 2d.

**
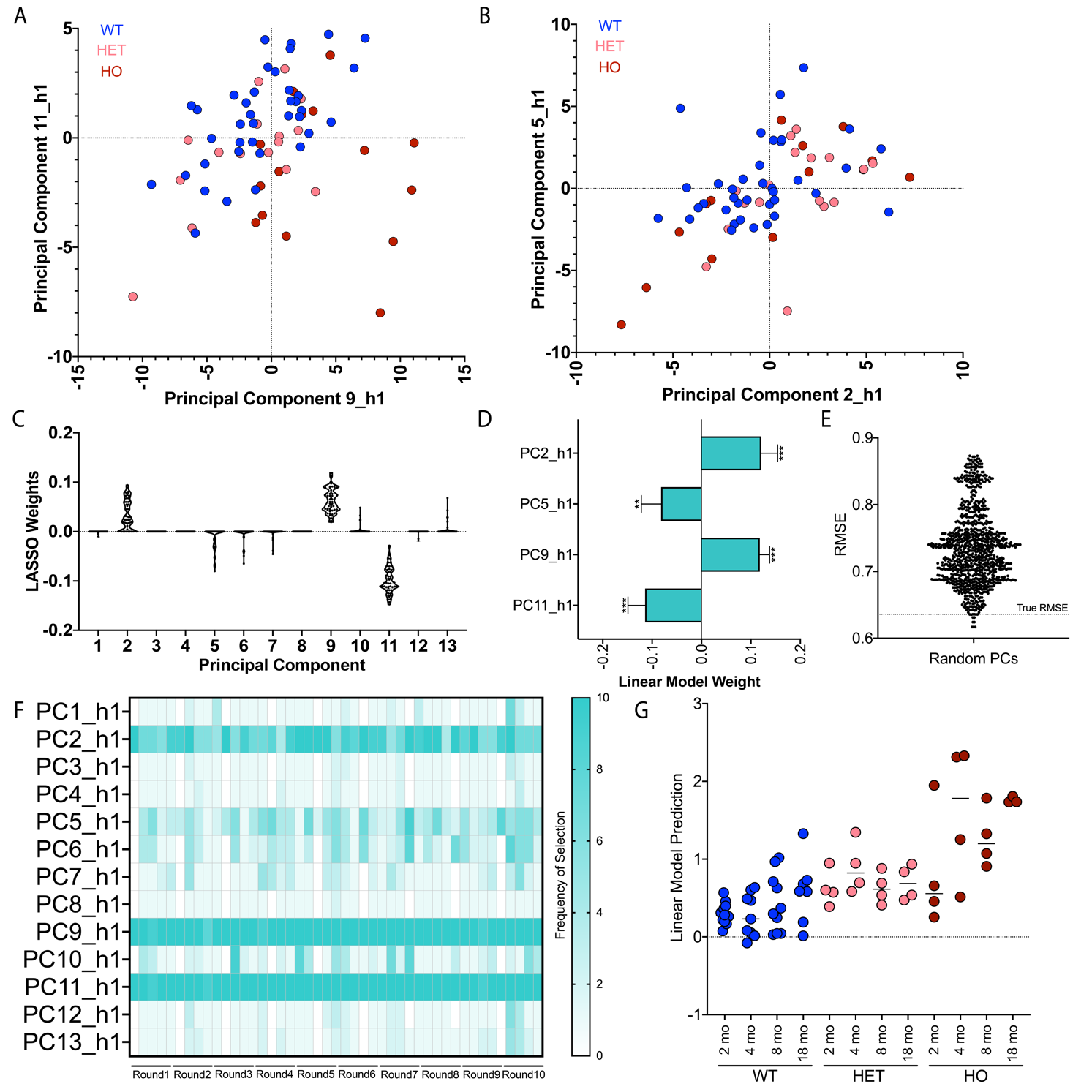
**

**Supplementary Figure 2. Additional modeling results from TransComp-R analysis of the first human cohort (GSE48350) and TASTPM mouse samples**

(A) Visualizing the mouse samples along PC9_h1 and PC11_h1, two of the PCs selected via LASSO.

(B) Visualizing the mouse samples along PC2_h1 and PC5_h1, two of the PCs selected via LASSO.

(C) LASSO weights from ten rounds of 5-fold cross from a representative feature selection run.

(D) Linear model weights for the four PCs selected via LASSO. Error bars represent SEM values.

(E) The final linear model was compared against null models. The RMSE of the true model had a p-value of 0.014 relative to null models that were generated using random PCs. Specifically, 1000 null models were generated using size-matched, random PCs.

(F) LASSO feature selection generates different results run-to-run. The heatmap shows the frequency of PC selection from ten separate LASSO runs.

(G) Visualizing linear model predictions for the final four-PC model with samples further stratified by sample age within each disease category

**
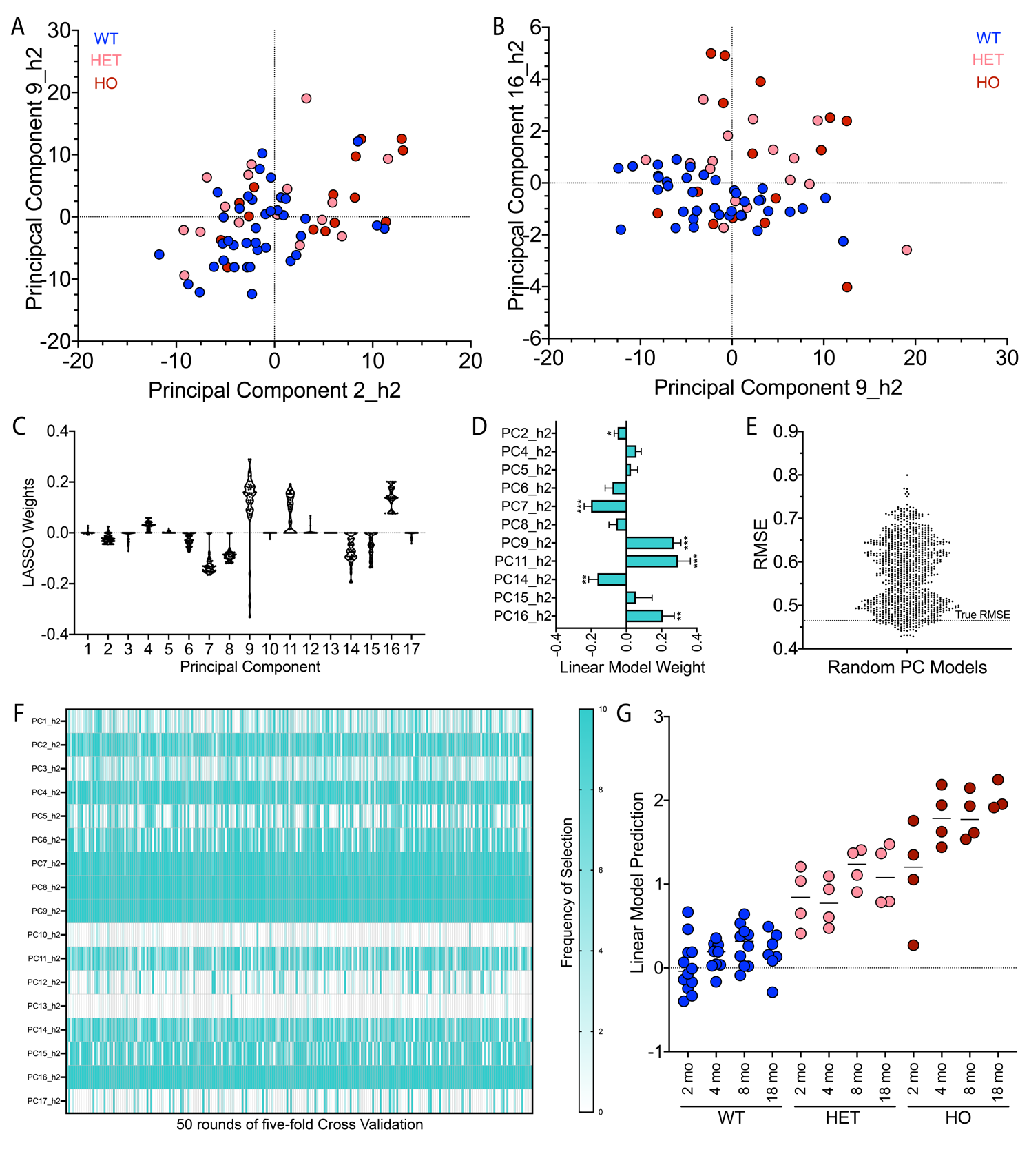
**

**Supplementary Figure 3. Additional modeling results from TransComp-R analysis of the second human cohort (GSE1297) and TASTPM mouse samples**

(A) Visualizing mouse samples along PC2_h2 and PC9_h2

(B) Visualizing mouse samples along PC9_h2 and PC16_h2

(C) LASSO weights from ten rounds of 5-fold cross validation from a representative feature selection run.

(D) Linear model weights for the final model. Error bars represent SEM values.

(E) The final linear model was compared against null models. The RMSE of the true model had a p-value of less than 0.067 relative to null models that were generated using random PCs.

(F) LASSO feature selection generates different results run-to-run. The heatmap shows the frequency of PC selection from fifty separate LASSO runs. The number of independent LASSO runs was increased from Supplementary Figure 2f to identify a predominant combination of PCs. The increased number of runs for certainty was necessary due to the increased number of components being selected near the 40 percent threshold.

(G) Visualizing linear model predictions for the final TransComp-R model with samples further stratified by sample age within each disease category

# Supplementary Tables (.xlsx)

**Table S1.** List of mSigDB pathways included in original statistical over-representation analysis

**Table S2.** List of mSigDB pathways included in secondary synapse-focused statistical over-representation analysis

**Table S3.** Pathway enrichment results for translatable components from second TransComp-R case study
